# Genome-Wide Identification and Characterization of Ammonium Transporter (AMT) Genes in Rapeseed (*Brassica napus* L.)

**DOI:** 10.3390/genes14030658

**Published:** 2023-03-06

**Authors:** Jing Dai, Peipei Han, Thomas C. Walk, Ling Yang, Liyu Chen, Yinshui Li, Chiming Gu, Xing Liao, Lu Qin

**Affiliations:** 1 Key Laboratory of Biology and Genetics Improvement of Oil Crops of the Ministry of Agriculture and Rural Affairs, Oil Crops Research Institute of Chinese Academy of Agricultural Sciences, Wuhan 430000, China; 2 Institute of Agriculture Science in Jiangsu Coastal Area, Yancheng 224002, China; 3 Tropotech LLC, St. Louis, MO 63141, USA; 4 Innovative Center of Molecular Genetics and Evolution, School of Life Sciences, Guangzhou University, Guangzhou 510006, China

**Keywords:** genome-wide analysis, expression profile, stress response

## Abstract

Ammonium transporters (AMTs) are plasma membrane proteins mediating ammonium uptake and transport. As such, AMTs play vital roles in ammonium acquisition and mobilization, plant growth and development, and stress and pathogen defense responses. Identification of favorable AMT genotypes is a prime target for crop improvement. However, to date, systematic identification and expression analysis of *AMT* gene family members has not yet been reported for rapeseed (*Brassica napus* L.). In this study, 20 *AMT* genes were identified in a comprehensive search of the *B. napus* genome, 14 members of *AMT1* and 6 members of *AMT2*. Tissue expression analyses revealed that the 14 *AMT* genes were primarily expressed in vegetative organs, suggesting that different *BnaAMT* genes might function in specific tissues at the different development stages. Meanwhile, qRT-PCR analysis found that several *BnaAMTs* strongly respond to the exogenous N conditions, implying the functional roles of *AMT* genes in ammonium absorption in rapeseed. Moreover, the rapeseed *AMT* genes were found to be differentially regulated by N, P, and K deficiency, indicating that crosstalk might exist in response to different stresses. Additionally, the subcellular localization of several BnaAMT proteins was confirmed in *Arabidopsis* protoplasts, and their functions were studied in detail by heterologous expression in yeast. In summary, our studies revealed the potential roles of *BnaAMT* genes in N acquisition or transportation and abiotic stress response and could provide valuable resources for revealing the functionality of *AMTs* in rapeseed.

## 1. Introduction

Nitrogen (N) is an essential macronutrient for plant growth and development that can be acquired as nitrate (NO_3_^−^), ammonium (NH_4_^+^), amino acids, and other N-containing substances [1]. In plants, NH_4_^+^ ions accumulating in cells, either through uptake from the soil via ammonium transporters (AMTs) or through reduction of NO_3_^−^, may be directed into the glutamine synthetase/glutamate synthase (GS/GOGAT) cycle [2]. Due to lower energy requirements for uptake and assimilation of NH_4_^+^ than NO_3_^−^, NH_4_^+^ is the preferred N source for uptake through roots, particularly in N-deficient plants [3,4]. On the other hand, excessive NH_4_^+^ concentrations are toxic and inhibit plant growth [5]. Thus, well-regulated homeostasis of internal NH_4_^+^ concentrations is essential for plant health and productivity.

Physiological studies of higher plants have revealed two transport systems for NH_4_^+^ on root cell membranes: a high-affinity ammonium transport system (HATS) and a low-affinity ammonium transport system (LATS). Under low external NH_4_^+^ concentrations, the HATS is upregulated for efficient absorption, while LATS products are more highly expressed at higher external NH_4_^+^ concentrations [6]. Coincidently, it is well known that plant *AMTs* are encoded by two distinct gene subfamilies: the *AMT1* subfamily (*AMT1* cluster) and the *AMT2* subfamily (*AMT2/3/4* clusters) [7]. *AMT1s* and *AMT2s* each typically contain 11–12 putative transmembrane regions, with two signature sequences located at transmembrane regions 5 and 10 [6,8].

To date, numerous *AMT* genes have been characterized in many plant species and are well documented in a published review [9], including *Arabidopsis* and rice. Previous studies found that *AMTs* have different expression characteristics within a plant species. For example, in *Arabidopsis thaliana*, *AtAMT1;1* is mainly expressed in roots and leaves, while *AtAMT1;2*, *AtAMT1;3*, *AtAMT1;5*, and *AtAMT2;1* are predominantly expressed in roots [10]. It was demonstrated that *AtAMT1;1* and *AtAMT1;3* account for 30–35% of NH_4_^+^ uptake in N-deficient roots [11], and *AtAMT1;2* accounts for 18–26% [12]. Additionally, *AtAMT1;4* is a pollen-specific ammonium transporter, mediating ammonium uptake across the plasma membrane of pollen, which contributes to N nutrition of pollen [13].

Ammonium is a major and preferential N form for rice grown in paddy fields due to poor aeration. It has been well documented that rice contains at least 12 *AMTs* [14], with these sequences being divisible into four subfamilies (*OsAMT1*, *OsAMT2*, *OsAMT3*, and *OsAMT4*), each of which is expressed in roots. Among these rice *AMT* subfamilies, *OsAMT1* members have been characterized as HATS transporters, while the other subfamilies contain only LATS transporters [15,16]. As well as *AtAMT1;1*, *OsAMT1;1* also exhibited ammonium uptake ability, with knockout of *OsAMT1;1* reducing the ammonium uptake capacity of rice by about 25–30% in vivo. Furthermore, this gene was mainly expressed in root stele, root and shoot vascular bundles, and leaf mesophyll cells. Knockout of *OsAMT1;1* resulted in a higher distribution of N in the root under low-NH_4_^+^ conditions [17], indicating that *OsAMT1;1* contributes to root-to-shoot ammonium translocation. Recently, research has found that knockout of *OsAMT1;1*, *OsAMT1;2*, and *OsAMT1;3* resulted in a 95% reduction in ammonium uptake, suggesting these three genes were cooperatively responsible for ammonium uptake under low-NH_4_^+^ conditions [18].

Besides the physiological roles of *AMTs* in mediating NH_4_^+^ acquisition from soil, root-to-shoot translocation of NH_4_^+^, and NH_4_^+^ uptake in leaves and the reproductive organs, *AMTs* also revealed roles in abiotic stress defense. It was reported that overexpression of *PutAMT1;1* promoted early root growth after seed germination in transgenic *Arabidopsis* under salt stress, suggesting that *AMT* could alleviate NH_4_^+^ toxicity caused by salt stress [19]. In addition, several *AMTs* were also related to drought stress, such as *AMT1;2* and *AMT1;6* upregulated in *Populus simonii* [20]. It is also found in *Malus prunifolia*. Two ammonium transporters (*AMT1;2* and *AMT4;2*) were notably upregulated together with the net influx of NH_4_^+^ at the surface of the roots under drought stress [21].

Rapeseed (*B. napus* L.) is one of the most essential and widely cultured oilseed crops worldwide for food and non-food purposes. In agriculture, rapeseed growth and yield require abundant N supplies [22,23]. Improving understanding of how uptake and transport of NH_4_^+^ and NO_3_^−^ are regulated in this genus might facilitate improved nutrient management in rapeseed crops, especially under N deficiency conditions. In this study, we isolated and characterized 20 *AMT* genes from a rapeseed genomic sequence. Subsequently, we comprehensively analyzed rapeseed AMT genes’ transcription profiles in various plant tissues subjected to NH_4_^+^ deficiency or sufficiency treatments. The distinct expression patterns of *BnaAMTs* might indicate the diverse physiological roles played by ammonium transporters in rapeseed. Overall, this genome-wide analysis of rapeseed *AMT* genes provides a basis for further investigation of these genes to identify specific valuable functions that can be selected to improve rapeseed productivity.

## 2. Materials and Methods

### 2.1. Plant Materials and Stress Treatments

A widely grown Chinese rapeseed cultivar, Zhongshuang 11 (*B. napus* cv. ZS11), was used in this study. This variety was bred by the Oil Crops Research Institute at the Chinese Academy of Agricultural Sciences (CAAS).

For tissue-specific expression analysis of *BnaAMT* genes, rapeseed plants were harvested at different developmental stages for RNAseq assays. Details regarding sample harvesting and RNAseq analysis were described by previous studies [24,25]. Rapeseed seedlings were cultured in normal N conditions for 10 days in hydroponics to analyze *BnaAMTs*’ responses to different forms of N supply. Seedlings were then transferred into N starvation conditions for 5 days, after which young rapeseed leaves, old leaves, and roots were separately collected at 1, 4, 8, 12, and 24 h after resupplying N-deficient rapeseed plants with NO_3_^−^ or NH_4_^+^. Samples were collected for each date and immediately stored at −80 °C for RNA extraction and subsequent qRT-PCR analysis.

To analyze the potential functions of *BnaAMT* genes in response to different nutrient deficiencies and drought stress, the experiments were conducted through simulation of these different stresses in hydroponics or a pot experiment as described before [26], respectively.

### 2.2. Identification and Bioinformatics Analyses of AMT Genes in Rapeseed

The amino acid sequences of all reported AMT members in *Arabidopsis*, rice, and wheat were used as query sequences to identify the *AMT* genes in rapeseed based on the *B. napus* genome database (http://www.genoscope.cns.fr/brassicanapus/ (accessed on 13 February 2023)). All potential proteins from the BLAST search were further filtered based on the presence of the conserved domain of AMT proteins (Pfam: PF00909) through an HMMER (3.1) search with the threshold value set at 0.001. The nucleotide and amino sequences of confirmed *BnaAMT* genes and their chromosomal locations were obtained from the *Brassica* database website. *AMT* Genes were then named according to their homologous genes in *Arabidopsis*. The distribution of *BnaAMT* genes on rapeseed chromosomes was plotted using the R package RIdeogram (https://github.com/TickingClock1992/RIdeogram (accessed on 13 February 2023)). Protein molecular weights and theoretical pI values were computed in the ProtParam tool (http://web.expasy.org/protparam/ (accessed on 13 February 2023)). Subcellular localization predictions for rapeseed AMT proteins were performed in ProtComp 9.0 (http://linux1.softberry.com/berry.phtml?group=programs&subgroup=proloc&topic=protcomppl (accessed on 13 February 2023)). Protein sequence alignment was performed using ClustalW and subsequently visualized in Genedoc. The logos of consensus transport residues were generated in WebLogo 3 online (http://weblogo.threeplusone.com/ (accessed on 13 February 2023)). The phylogenetic tree was constructed based on protein sequence alignment of AMT family sequences through the neighbor-joining method with 1000 bootstrap replicates in the MEGA 7.0 program (http://www.megasoftware.net/download_form (accessed on 13 February 2023)). The CDS and genomic sequences of rapeseed *AMT* genes downloaded from the database were used to paint gene structures through the use of Gene Structure Display Server 2.0 (http://gsds.cbi.pku.edu.cn (accessed on 13 February 2023)).

### 2.3. Expression Analysis

Total RNA from different rapeseed samples was extracted using RNAiso^TM^ Plus reagent (Takara Bio, Otsu, Shiga, Japan) according to the manufacturer’s manual. RNA samples were then purified with RNase-free DNaseI (Invitrogen, Grand Island, NY, USA) to remove any contaminating genomic DNA. Total RNA quality assessment was checked via NanoDrop^®^ spectrophotometry (TGem Plus, Tiangen, Beijing, China) and agarose gel electrophoresis to confirm the 28S:18S rRNA ratio. Next, first-strand cDNA sequences were synthesized using the PrimeScript™ RT Master Mix (Takara, Tokyo, Japan) according to the manufacturer’s protocols. The synthesized cDNA was used for forqRT-PCR reactions in a CFX connect Real-Time PCR Detection System (Bio-Rad, Hercules, CA, USA) using the SYBR^®^ Premix Ex Taq™ II (TaKaRa, Tokyo, Japan). The related primers for qRT-PCR are listed in Table S1 and were designed with Primer-NCBI (https://www.ncbi.nlm.nih.gov/tools/primer-blast/index.cgi?LINK_LOC=BlastHome (accessed on 13 February 2023)). The PCR reactions were performed according to the manual of the SYBR^®^ Premix Ex Taq™ II and with a total volume of 20 μL under the following conditions: 95 °C for 1 min, followed by 40 cycles of 95 °C for 15 s, 60 °C for 15 s, and 72 °C for 30 s. The expression of each *AMT* gene was calculated by the method described before and normalized to *actin 7* [27,28]. Four biological replicates were used for each measurement.

### 2.4. Yeast Mutant Complementation Analysis

Full-length cDNA sequences of *BnaAMT1;1b*, *BnaAMT1;1c*, *BnaAMT1;4a*, *BnaAMT1;5a*, and *BnaAMT2;2a* were amplified by PCR using specific primers containing the HindIII, XbaI, or KpnI sites, as listed in Table S2. Returned open reading frames were ligated into the yeast expression vector pYES2 after the vector was linearized by HindIII, XbaI, or KpnI digestion. Yeast *Δmep1, 2, 3* mutant 31019b, which cannot grow with <5 mM NH_4_^+^ provided as the sole N source [29], was transformed with pYES2-*BnaAMT1;1b*, pYES2-*BnaAMT1;1c*, pYES2-*BnaAMT1;4a*, pYES2-*BnaAMT1;5a*, or pYES2-*BnaAMT2;2a*, with the empty vector pYES2 also included as a negative control. All transformants were first selected on a solid yeast nitrogen base medium (2% agar) supplemented with 2% D-galactose and 2 mM L-arginine as the N source. A single colony was then picked, suspended in 100 mL of water, serially diluted, and dropped (2 µL) onto solid SD medium supplemented with 2% D-galactose and 0.02, 0.2, 2, or 5 mM NH_4_Cl provided as the sole N source, with pH values adjusted to 5.8 as described before with slight modification [30].

### 2.5. Subcellular Localization of Rapeseed AMT Proteins

Five representative BnaAMT proteins were cloned to generate constructs for subcellular localization analysis in *Arabidopsis* protoplasts. The ORF of each *BnaAMT* gene was amplified for insertion into the pMDC43 vector with *Hind*III/*Kpn*I and *Xba*I to generate BnaAMT-GFP fusion proteins driven by the CaMV 35S promoter. The gene-specific primers are listed in Table S2. The vectors were respectively transformed into *Arabidopsis* protoplasts, which were isolated from 4-week-old leaves according to previous work [31]. After transfection and incubation in a plate under weak light for 12–16 h, fluorescent cells were imaged using a laser scanning confocal microscope (OLYMPUS FV10-ASW, Olympus, Tokyo, Japan).

### 2.6. Statistical Analyses

All data were analyzed in Microsoft Excel 2010. The comparisons were performed between the means of control and stress treatments using the one-way analysis of variance (ANOVA) method at the 5% and 1% probability level in SPSS statistics 25.

## 3. Results

### 3.1. Identification of AMT Genes in Rapeseed

**Figure 1:**
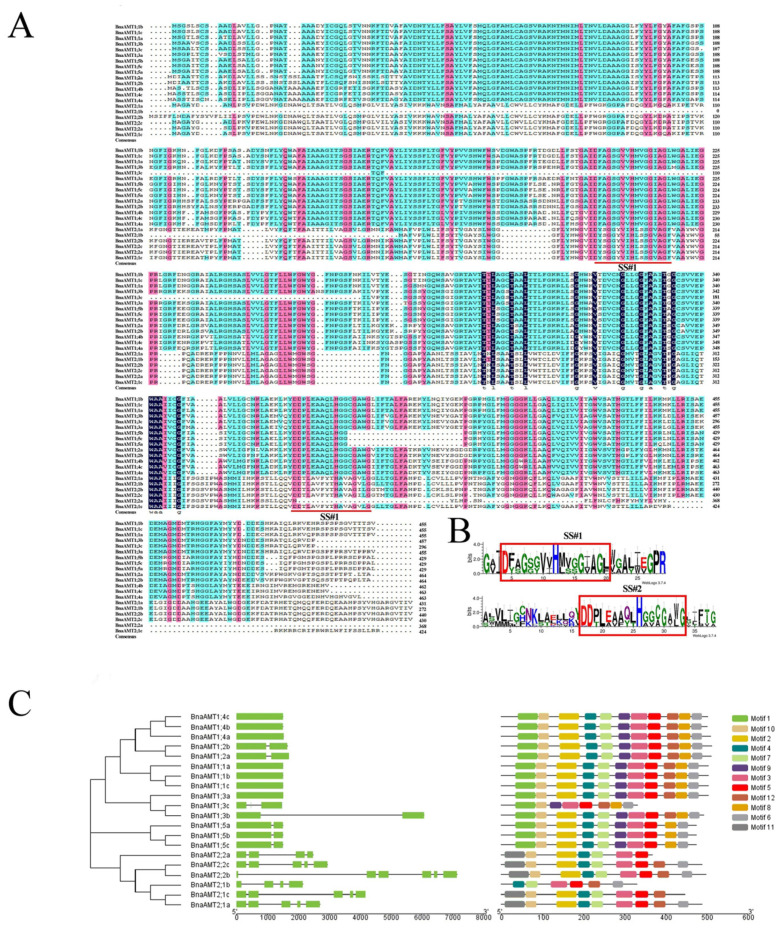
Multiple alignment of rapeseed AMT family proteins and gene structure/conserved motifs characteristic of rapeseed AMT family members. (**A**) Multiple alignment of amino sequences of BnaAMTs. Red lines underneath alignments indicate two reported signature sequences within the AMT family. (**B**) The logo of these signature sequences. (**C**) The gene structure map of *BnaAMTs*. Green boxes indicate exons while black lines represent introns. The conserved motifs of rapeseed AMT proteins were analyzed through MEME.

Potential *AMT* genes were first identified by BLAST searching the *B. napus* genome with *AMT* sequences from *Arabidopsis*, rice, and wheat. The potential *B. napus AMTs* were checked for the ammonium transporter family Pfam domain (‘PF00909′) to further narrow down rapeseed *AMT* genes. Ultimately, 20 putative AMT proteins and their encoding genes were identified from the *B. napus* genome. These genes were named based on the order of homologies from *Arabidopsis*. The length of encoded proteins ranged from 447 amino acids (a.a.) to 512 a.a., with 7 to 11 transmembrane regions included in each protein sequence (Table 1 and Figure 1A) and two signature sequences located at transmembrane domains 5 and 10 (Figure 1B). All the putative proteins identified were predicted to localize to the plasma membrane (Table 1). The conserved motifs in the AMT protein sequences were predicted by MEME in rapeseed (Figure 1C). The BnaAMT proteins in the same subgroup showed identical motif components. Motifs 1–10 were commonly identified in all members of the AMT1 subgroup, except for *BnaAMT1;3c*. However, Motif 12 seemed to be distributed explicitly in the AMT2 subgroup.

### 3.2. Phylogenetic Analyses and Chromosomal Locations of BnaAMT Genes

**Figure 2:**
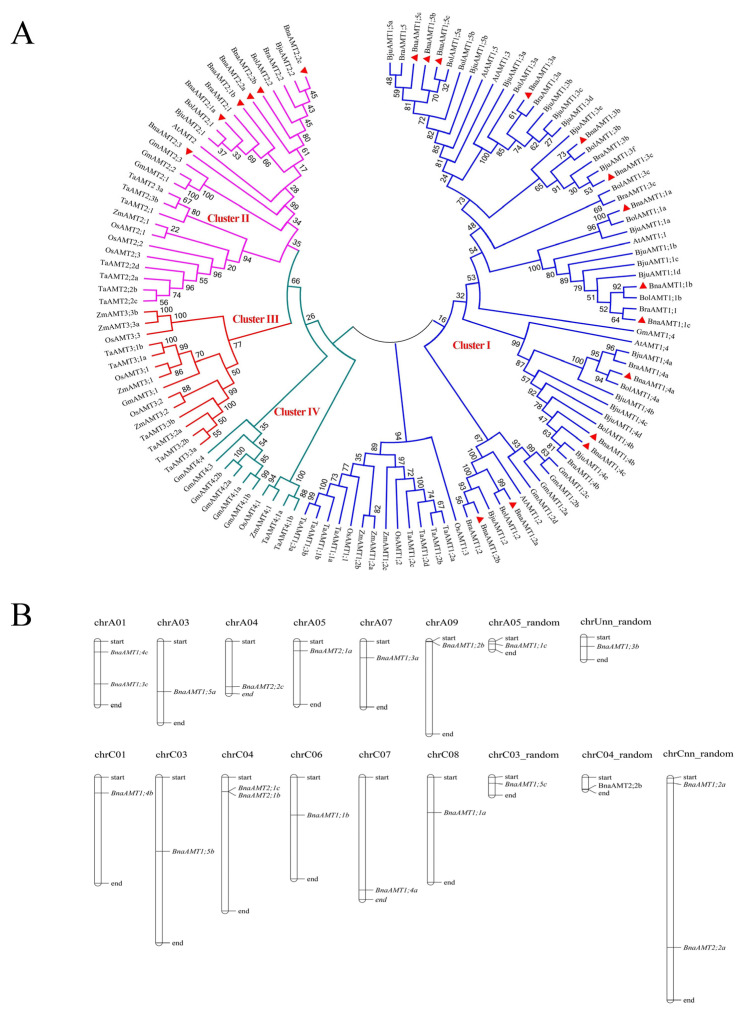
Phylogenetic relationships of AMT family proteins in diverse species and the chromosomal locations of *AMT* family genes in rapeseed. (**A**) Phylogenetic tree of AMT family proteins. A total of 125 proteins from nine species were used to construct a phylogenetic tree based on the neighbor-joining method in MEGA 7.0 (1000 replicates). From previous reports, 39 AMT family proteins from *A. thaliana* (At), *Oryza sativa* (Os), and *Triticum aestivum* (Ta) were identified. The AMT family proteins from *B. napus* (Bna), *Glycine max* (Gm), *Zea mays* (Zm), *B. rape* (Bra), *B. oleracea* (Bol), and *B. juncea* (Bju) were predicted from these 39 known AMT proteins. Different clusters are labeled by different colors. The 20 aligned BnaAMTs are marked by red triangles. (**B**) Positions of *AMT* family genes on rapeseed chromosomes.

To evaluate evolutionary relationships among orthologous *AMT* genes, a phylogenetic tree of AMTs from rapeseed, *Arabidopsis*, soybean, rice, corn, wheat, and three *Brassica* relatives of rapeseed was constructed using the neighbor-joining method in MEGA 7.0. As shown in Figure 2A, two major clades and four clusters are distinguishable in this phylogenetic tree. Among the 20 *AMT* genes in rapeseed, 14 fell into the AMT1 cluster and 6 were found in the AMT2 cluster. No rapeseed or other brassica *AMT* genes were placed into either of the two remaining clusters (AMT3 and AMT4). The chromosomal locations of rapeseed AMT genes are also shown in Figure 1. These *BnaAMT* genes are scattered across the *B. napus* genome, with the only cluster found containing *BnaAMT2;1b* and *BnaAMT2;1c* on chromosome C04 (Figure 2B).

### 3.3. Expression Patterns of BnaAMT Genes in Various Rapeseed Tissues

**Figure 3:**
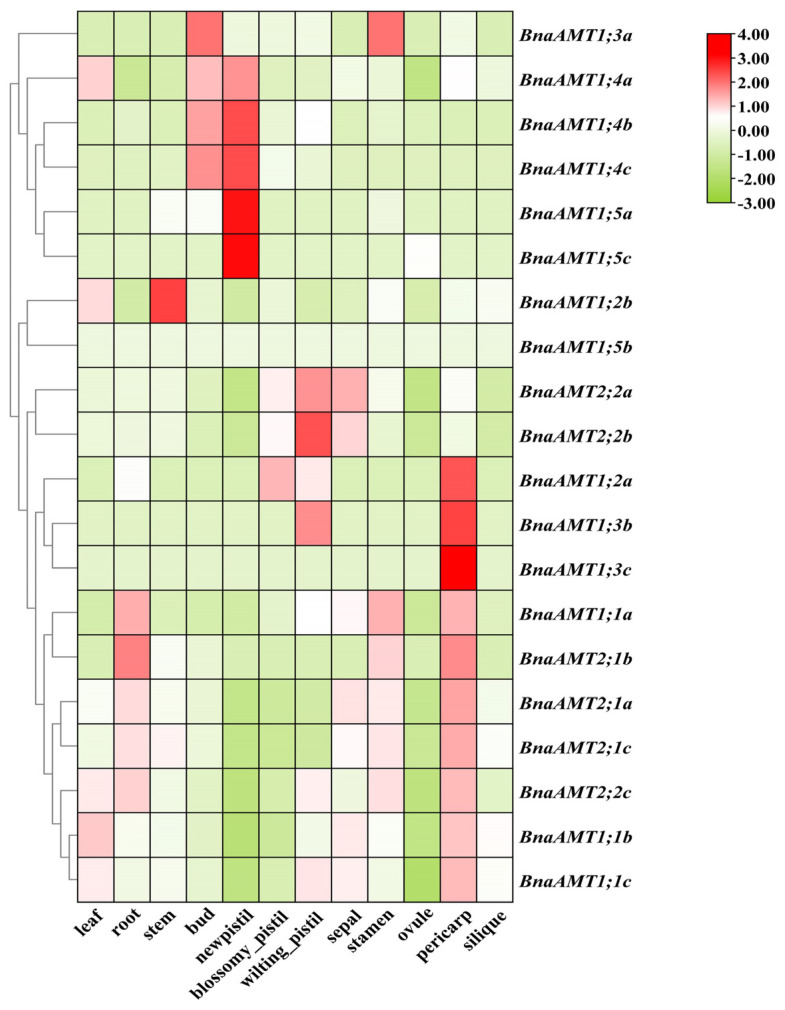
Tissue-specific expression profiles of *BnaAMT* family genes. Heat maps of *BnaAMT* expression were generated from RNA-seq analysis in twelve different tissues, including leaves, roots, stems, buds, new pistils, blossomy pistils, wilting pistils, sepals, stamens, ovules, pericarps, and siliques. The red color indicates upregulation, while the green color indicates downregulation.

For indications of the biological functions filled by *BnaAMT* genes, the expression patterns of these genes were determined in different tissues through RNA-seq analysis. Among the 20 *AMT* genes examined, two (*BnaAMT1;5b* and *BnaAMT1;5c*) were not detected in any tissue under the applied growth conditions, and eight exhibited relatively low expression levels in a majority of tissues (Figure 3). Expression of the remaining half of *BnaAMT* genes, though variable, was relatively high in most tissues. *BnaAMT1;1b*, *BnaAMT1;1c*, *BnaAMT2;1a*, and *BnaAMT2;1c* were widely expressed across most tissues, both aboveground and belowground, including vegetative and reproductive organs. *BnaAMT1;4a*, *BnaAMT1;4b*, and *BnaAMT1;4c* were most highly expressed in new pistil and bud tissues. In addition, BnaAMT2;2a was also interesting for being expressed at high levels in sepals and blossomy pistils (Figure 3). Finally, under the conditions of this experiment, no *BnaAMT* genes were found to be specifically expressed in roots.

### 3.4. Expression of BnaAMT Genes in Response to N Deficiency and Resupply

**Figure 4:**
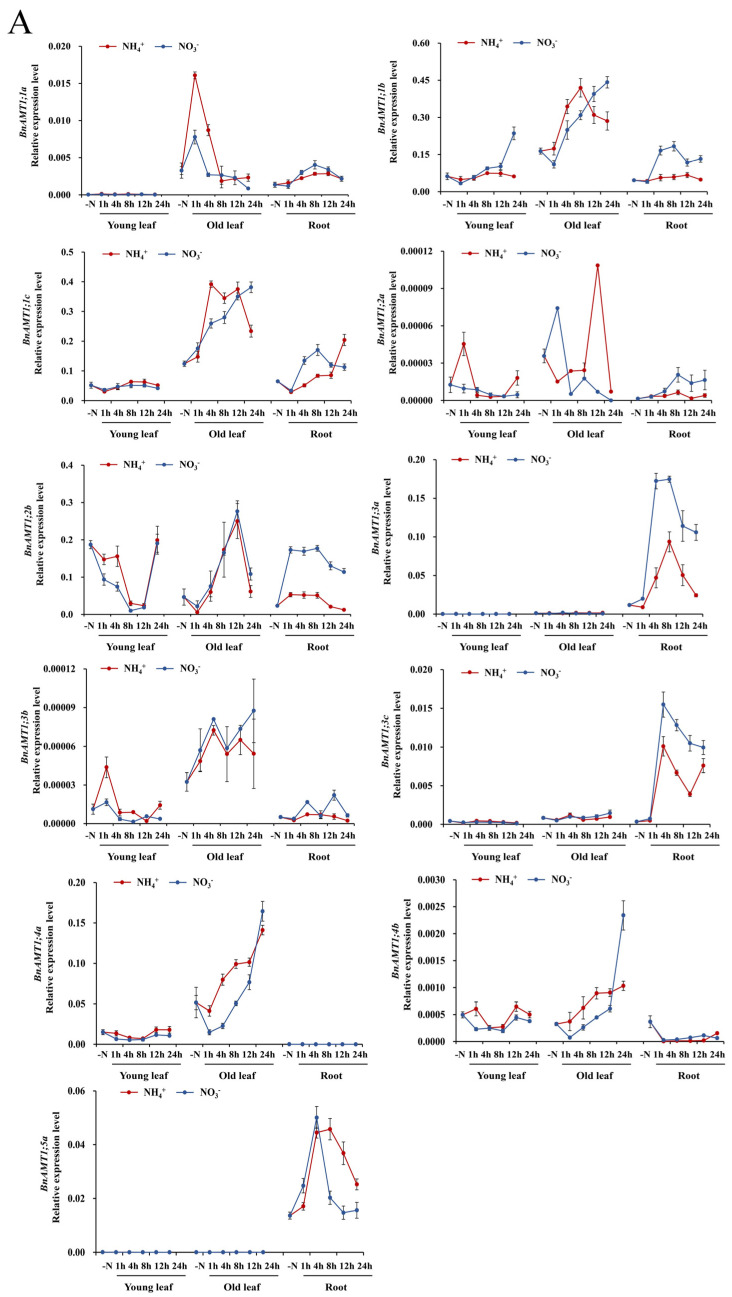

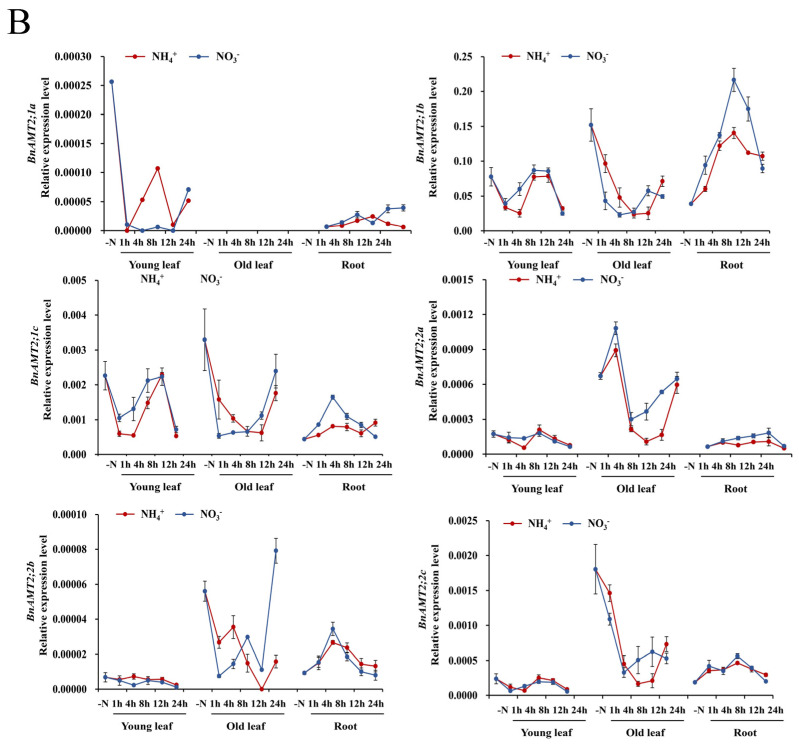
The expression profiles of *BnaAMTs* in different tissues of rapeseed with ammonium and nitrate supply. (**A**) *BnaAMT1s*; (**B**) *BnaAMT2s*. The red lines indicate ammonium source, while blue lines indicate nitrate. Rapeseed young leaves, old leaves, and roots were separately collected at 1 h, 4 h, 8 h, 12 h, and 24 h after resupplying N-deficient rapeseed plants with NH_4_^+^ or NO_3_^−^. Samples were stored at −80 °C for RNA extraction and qRT-PCR analysis. The relative expression levels of *BnaAMTs* were relative to the control (*actin 7*). Four biological replicates were performed. Error bars represent standard deviation.

To test whether any *BnaAMT* responses to N deficiency were mediated by NH_4_^+^ or NO_3_^−^, expression of *BnaAMTs* was examined in 10-day-old rapeseed seedlings grown under N deficiency conditions for 5 days and then resupplied with 1 mM NH_4_^+^ or 1 mM NO_3_^−^ for 0, 1, 4, 8, 12, and 24 h. Within 1 h of initiating ammonium resupply, *BnaAMT1;1a* and *BnaAMT2;2a* mRNA levels increased in old leaves and then quickly peaked before receding to previous levels or even lower (Figure 4). Interestingly, while expression of *BnaAMT1;1b* and *BnaAMT1;1c* was similar to expression of *BnaAMT2;1a* and *BnaAMT2;1c* across tissues (Figure 3), their responses to NH_4_^+^ and NO_3_^−^ resupply treatments contrasted according to their subfamily membership (Figure 4). *BnaAMT1;1b* and *BnaAMT1;1c* mRNA levels increased in the old leaves of N-deficient rapeseed resupplied with either NH_4_^+^ or NO_3_^−^, though responses peaked temporarily in hours 4–12 of NH_4_^+^ resupply treatments and continued increasing for the full 24 h of observations when NO_3_^−^ was resupplied (Figure 4). In contrast, both *BnaAMT2;1a* and *BnaAMT2;1c* were downregulated in response to NH_4_^+^ or NO_3_^−^ resupply treatments in young leaves and old leaves, but not in roots. Notably, *BnaAMT2;1b* expression strongly declined in old leaves with ammonium or nitrate resupplies, but its expressions in roots increased significantly, especially after 8 h of nitrate resupply (Figure 4).

Among the remaining *BnaAMT* genes, the expression of several, including *BnaAMT1;2a*, *BnaAMT1;2b*, *BnaAMT1;4a*, and *BnaAMT1;4b*, peaked 12 h to 24 h after being resupplied with NH_4_^+^ or NO_3_^−^. Three *BnaAMT* genes, *BnaAMT1;3a*, *BnaAMT1;3c*, and *BnaAMT1;5a*, were detected almost exclusively in roots, where they all displayed transient peaks in expression 4–8 h after the onset of NH_4_^+^ or NO_3_^−^ resupply treatments (Figure 4).

### 3.5. Quantitative RT-PCR Analysis of BnaAMT Genes in Nutrient-Deficient Rapeseed Plants

**Figure 5:**
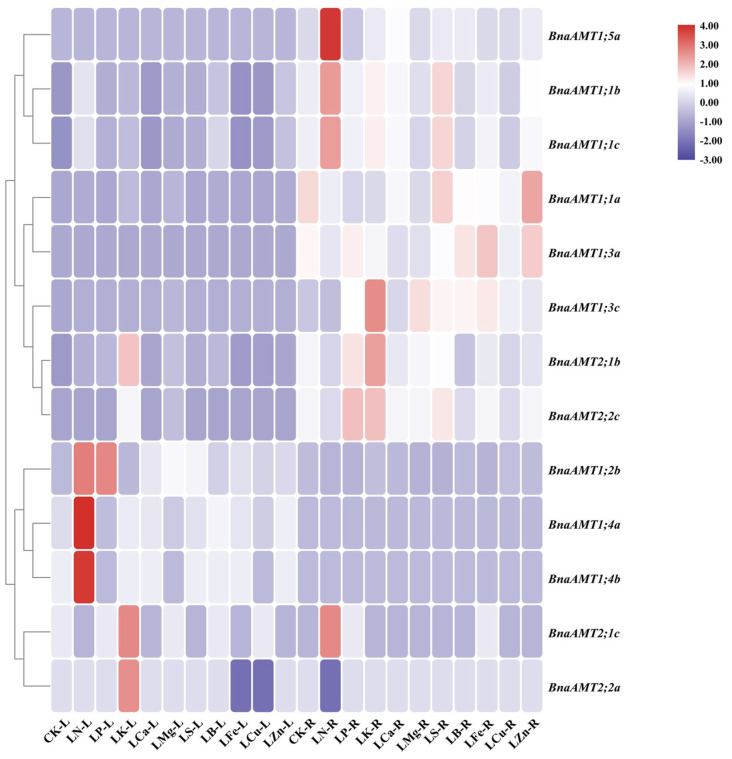
The relative expression profiles of *BnaAMT* family gene responses to different nutrient deficiencies. LN, N deficiency; LP, P deficiency; LK, K deficiency; LCa, Ca deficiency; LMg, Mg deficiency; LS, S deficiency; LB, B deficiency; LFe, Fe deficiency; LCu, Cu deficiency; LZn, Zn deficiency; CK, control. L, leaf; R, root. The red color indicates upregulation, while the blue color indicates downregulation. The relative expression levels of *BnaAMTs* were relative to the control (*actin 7*). Four biological replicates were performed.

Beyond potential functions in acquiring transient supplies of N, *BnaAMTs* might also be involved in responses to nutrient deficiency. To test this, expression of *BnaAMT* members was quantified in the roots and leaves of rapeseed plants subjected to deficiency in N, phosphorus (P), potassium (K), calcium (Ca), magnesium (Mg), sulfur (S), and boron (B). Among the 20 identified *BnaAMT* genes, the expression of 7 *BnaAMTs* was either not detected or was detected with very low levels of expression in the roots or leaves under any of the control or nutrient deficiency conditions. Rapeseed leaves responded to N deficiency by upregulating eight *BnaAMT* genes and downregulating one, while roots responded to N deficiency by upregulating five BnaAMT genes. Under P deficiency stress, only three *BnaAMT* genes were upregulated in leaves or roots, and five were remarkably depressed. In K-deprived rapeseed, nine *BnaAMT* genes were upregulated in leaves or roots, while only two exhibited significant decreases in abundance in K-deficient leaves. Interestingly, expression of *BnaAMT1;1a*, *BnaAMT1;2b*, and *BnaAMT1;3c* increased in the leaves of rapeseed in nearly all of the nutrient deficiency treatments, while only *BnaAMT1;3c*, being significantly upregulated in the roots of six of the nutrient deficiency treatments, responded consistently in roots across nutrient deficiency treatments (Figure 5).

### 3.6. Expression of BnaAMT Genes in Drought- or Waterlogging-Stressed Rapeseed

Drought and waterlogging are two common stresses in rapeseed production systems. To date, little is known about the expression profiles of *BnaAMT* genes in response to these two stresses. Therefore, the expression of 17 detectable *BnaAMT* transcripts was examined by qRT-PCR analysis under drought and waterlogging stress conditions. As in the nutrient deficiency experiment described above, *BnaAMT1;4c*, *BnaAMT1;5b*, and *BnaAMT1;5c* showed no expression. In addition, *BnaAMT1;3b* and *BnaAMT1;5a* exhibited only shallow expression in this drought and waterlogging experiment, so they were also excluded from this analysis. Therefore, results from waterlogging and drought stress trials are presented for the remaining 15 *BnaAMT* genes.

**Figure 6:**
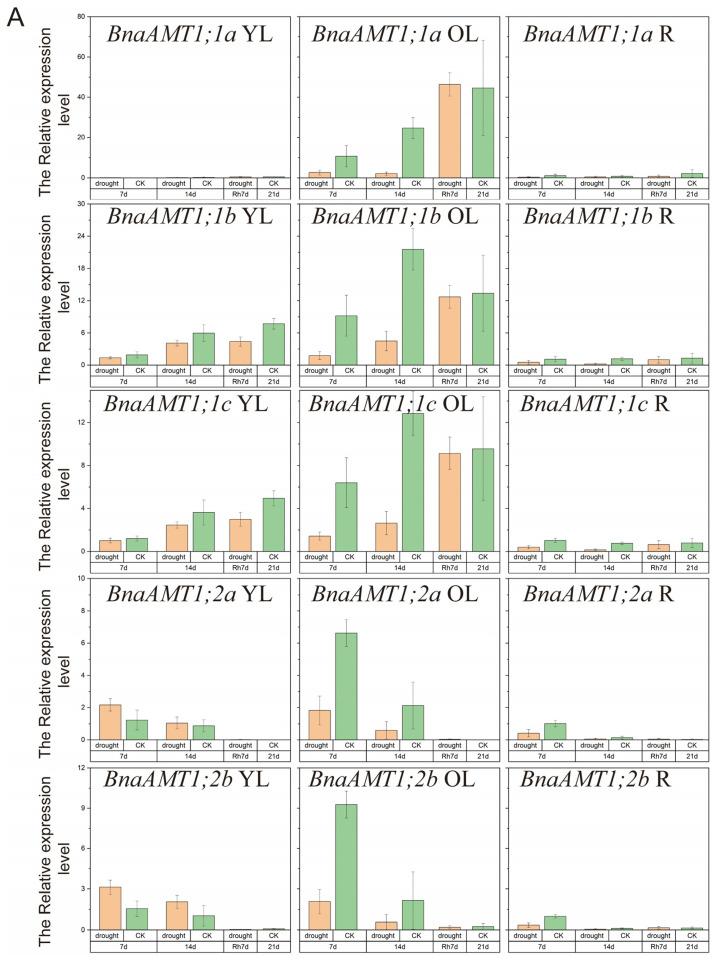

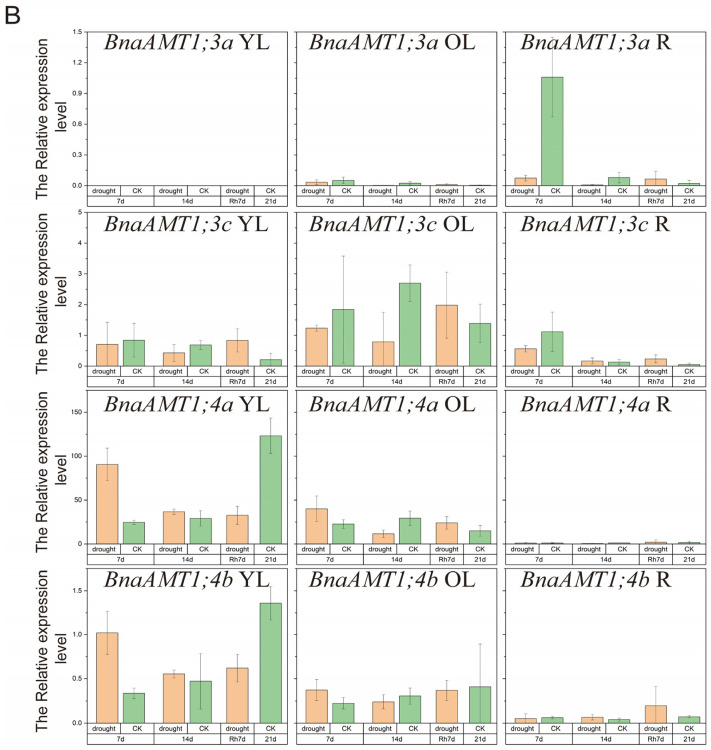

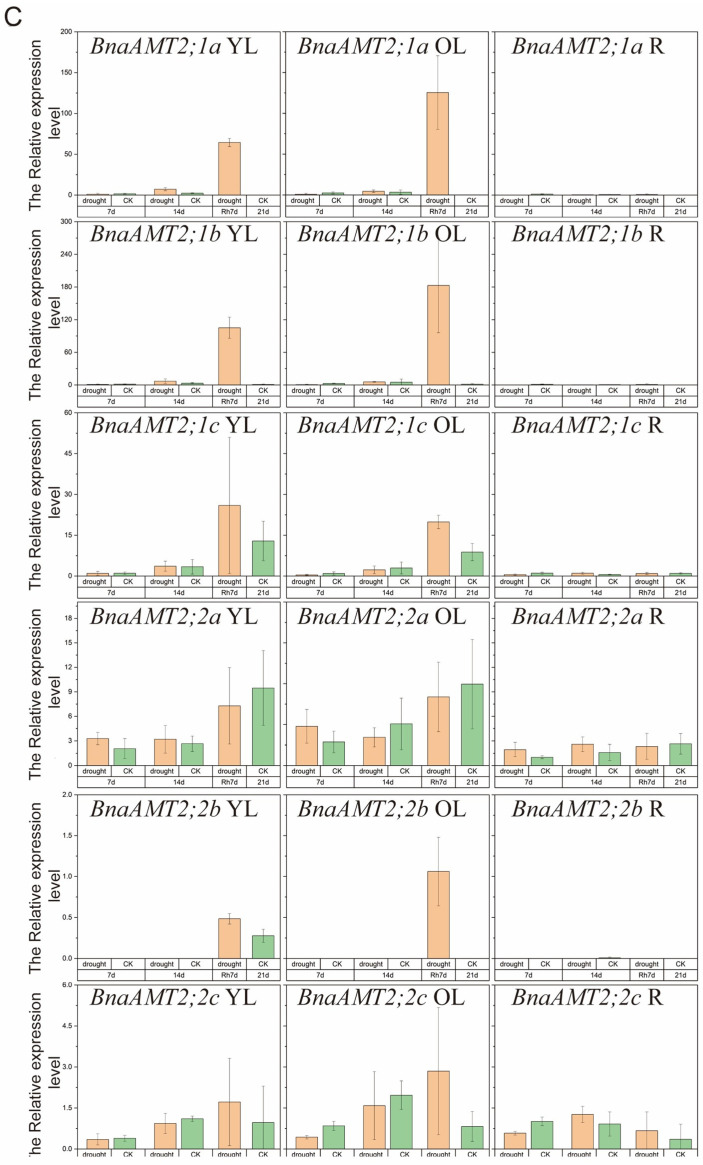
Expression profiles of *BnaAMT* family genes responding to drought stress. (**A**) The relative expression level of *BnaAMT1;1a*-*BnaAMT1;2b*; (**B**) the relative expression level of *BnaAMT1;3a*-*BnaAMT1;4b*; (**C**) the relative expression level of *BnaAMT2;1a*-*BnaAMT2;2c*. YL, young leaf; OL, older leaf; R, root; CK, control; drought, drought stress treatment; 7d, 7th day of drought stress; 14d, 14th day of drought stress; Rh7d, 7th day after rehydration; 21d, 21st day of drought stress. Rapeseed seedlings at the five-leaf growth stage were subjected to drought stress for 14 days before rehydration. The relative expression levels of *BnaAMTs* were relative to the control (*actin 7*). Four biological replicates were performed. Error bars represent standard deviation.

Under drought stress conditions, *BnaAMT* gene expression could be divided into four categories, as shown in Figure 6. Expressions of *BnaAMT1;2a*, *BnaAMT1;2b*, and *BnaAMT1;3a* were inhibited by drought stress in older leaves and roots. Several *BnaAMT* genes were inhibited by drought stress upon rehydration (rather than demonstrating a recovery in expression levels), including *BnaAMT1;1a*, *BnaAMT1;1b*, *BnaAMT1;1c*, *BnaAMT1;3c*, and *BnaAMT2;2a*. Transcription levels of *BnaAMT2;1a*, *BnaAMT2;1c*, *BnaAMT2;2b*, and *BnaAMT2;1b* increased dramatically with rehydration, especially in older leaves of rapeseed. In contrast to those downregulated *BnaAMT* genes, the expression of *BnaAMT1;2a*, *BnaAMT1;2b*, *BnaAMT1;4a*, and *BnaAMT1;4b* was significantly enhanced in young leaves after 7 days of drought stress.

**Figure 7:**
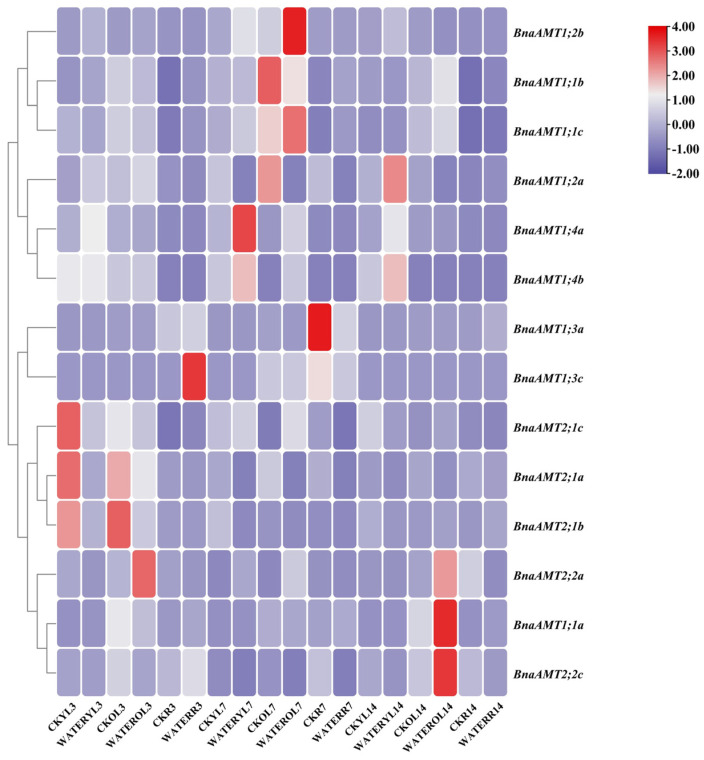
The relative expression profiles of *BnaAMT* family gene responses to waterlogging stress. CK, control treatment; WATER, waterlogging stress treatment; YL, young leaf; OL, old leaf; R, root; 3, after 3 days of treatment; 7, after 7 days of treatment; 14, after 14 days of treatment. The red color indicates upregulation, while the blue color indicates downregulation. The relative expression levels of *BnaAMTs* were relative to the control (*actin 7*). Four biological replicates were performed.

Under waterlogging stress conditions, the expression of several *BnaAMT* genes was inhibited by waterlogging stress, especially in early stress-treated leaves, such as three *BnaAMT2;1* members (Figure 7). Additionally, the expression of a number of *BnaAMTs* was remarkably enhanced in response to waterlogging stress in older leaves, namely the transcript of *BnaAMT1;1c* and *BnaAMT1;2b*, which were strongly upregulated after 7 days treatment, as well as the transcript of *BnaAMT1;1a* and *BnaAMT2;2c*, which dramatically increased with treatment after 14 days (Figure 7). These results implied that waterlogging stress might disrupt N metabolism in plants, and *AMT* genes might participate in translocating NH_4_^+^ to regulate N status in stressed plants.

### 3.7. Functional Complementation Analysis of Selected BnaAMT Genes in a Yeast Mutant Strain

**Figure 8:**
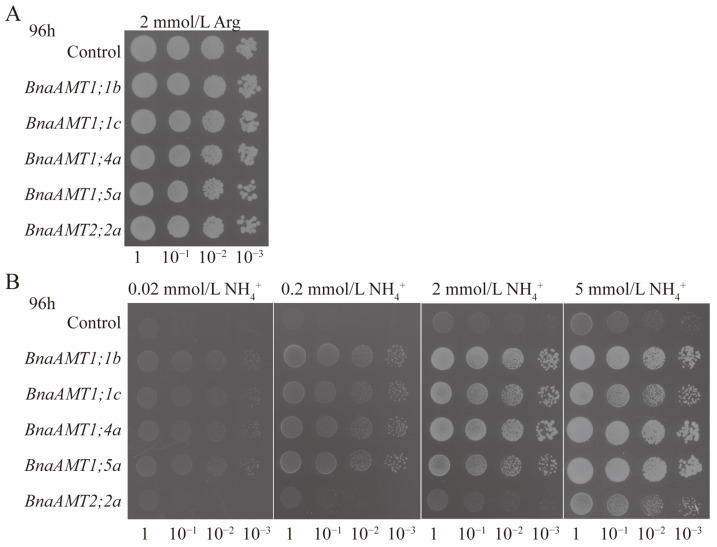
Functional complementation of the yeast mutant 31019b defective in NH_4_^+^ uptake by heterologous expression of *BnaAMTs* (*BnaAMT1;1b*, *BnaAMT1;1c*, *BnaAMT1;4a*, *BnaAMT1;5a*, *BnaAMT2;2a*). (**A**) The 31019b cells carrying the empty vector pYES2 (control), *BnaAMT1;1b*, *BnaAMT1;1c*, *BnaAMT1;4a*, *BnaAMT1;5a*, and *BnaAMT2;2a* were allowed yeast growth on 2 mM L-arginine as a sole N source. (**B**) The 31019b cells carrying the empty vector pYES2 (control), *BnaAMT1;1b*, *BnaAMT1;1c*, *BnaAMT1;4a*, *BnaAMT1;5a*, and *BnaAMT2;2a* were allowed yeast growth on 0.02, 0.2, 2, and 5 mM NH_4_Cl as a sole N source.

To rapidly test the putative NH_4_^+^ transport roles filled by *BnaAMTs*, the ORFs of *BnaAMT1;1b*, *BnaAMT1;1c*, *BnaAMT1;4a*, *BnaAMT1;5a*, and *BnaAMT2;2a* were separately cloned into lines of the yeast expression vector pYES2 and then transformed into yeast Δ*mep1, 2, 3* mutant 31019b, which cannot grow on media containing less than 5 mM NH_4_^+^ as the sole N source. Yeast 31019b cells carrying *BnaAMT1;1b*, *BnaAMT1;1c*, *BnaAMT1;4a*, *BnaAMT1;5a*, *BnaAMT2;2a*, or the empty pYES2 vector as control were all able to proliferate on yeast growth medium with 2 mM L-arginine provided as the sole N source (Figure 8A). Transformation with the empty vector pYES2 or pYES2 harboring *BnaAMT2;2a* did not stimulate growth on the medium containing up to 5 mM NH_4_^+^ (supplied as NH_4_Cl) as the sole source of N, while the transformation of 31019b with pYES2 harboring *BnaAMT1;1b*, *BnaAMT1;1c*, *BnaAMT1;4a*, and *BnaAMT1;5a* allowed yeast growth on media containing as little as 0.02 mM NH_4_Cl as a sole N source, with increasing NH_4_^+^ leading to more growth (Figure 8B). On the whole, results of yeast transformation indicate that *BnaAMT1;1b*, *BnaAMT1;1c*, *BnaAMT1;4a*, and *BnaAMT1;5a* facilitate NH_4_^+^ permeation across the plasma membrane, while *BnaAMT2;2a* may not function in this capacity.

### 3.8. Subcellular Localization of BnaAMT Proteins

**Figure 9:**
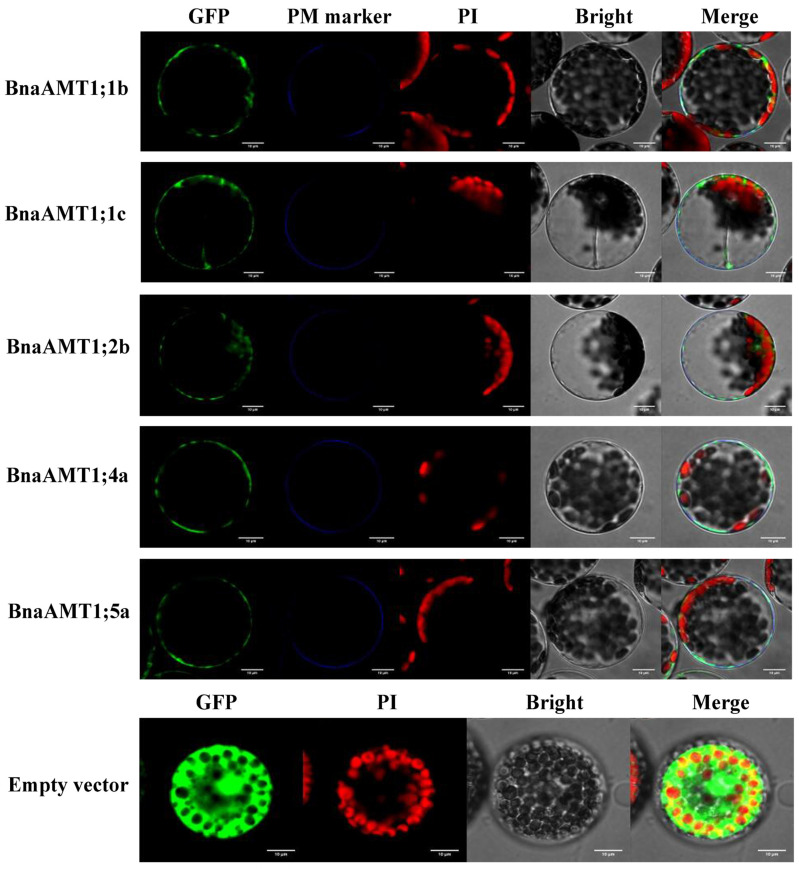
Subcellular localization of five representative BnaAMTs in *A. thaliana* protoplasts. OsMCA1, as the plasma membrane marker with the blue signals (PM Marker), was co-introduced into *A. thaliana* protoplasts with *BnaAMT* ORFs fused with GFP. An empty vector was included as a control. GFP, green fluorescence protein (green signals); PI, propidium iodide (red signals); Bright, bright field; Merge, overlay of all signals. White bars equal 10 μ0.

To explore the subcellular localization of the *BnaAMT* proteins, we first explored subcellular localization as predicted by ProtComp analysis. As shown in Table 1, all of the identified BnaAMT proteins were predicted to target plasma membranes. This was followed by experimental observations of the subcellular localizations of five selected BnaAMT proteins (BnaAMT1;1b, BnaAMT1;1c, BnaAMT1;4a, BnaAMT1;5a, and BnaAMT2;2a) through the transient expression of GFP::BnaAMT fusions in *Arabidopsis* protoplast cultures expressing the membrane marker OsMCA1. Microscopic observation revealed that each of the 35GFP::BnaAMT fusion constructs localized to plasma membranes along with OsMCA1 (Figure 9). These results strongly suggest that BnaAMT proteins consistently localize to plasma membranes, where they fulfill specific biological functions in rapeseed cells.

## 4. Discussion

Ammonium transporters play vital roles in ammonium uptake and translocation [1,4]. The *AMT* gene family has been investigated and characterized in various plant species, including rice, wheat, maize, cassava, and poplar [6,32,33,34,35]. Nevertheless, information on the *AMT* gene family remains lacking for rapeseed, a widely cultivated oil crop. Moreover, rapeseed is sensitive to N deficiency as an allotetraploid crop resulting from hybridization between *B. rapa* and *B. oleracea* [36]. Maintenance of productivity in rapeseed crops requires relatively large inputs of N fertilizer. Therefore, characterization of *AMT* members in rapeseed is a promising avenue to explore for improvements in the nutrient management of rapeseed or the targeting of traits in breeding programs aiming to produce rapeseed varieties with stronger tolerance to low N availability.

In this study, we comprehensively identified and characterized the *AMT* gene family in rapeseed, i.e., *B. napus*, which included analyzing the information of *AMT* homologous sequences in the *B. napus* genome, such as phylogenetic relationships, chromosome locations, gene structures, conserved motifs, and cis-acting promoter elements. Furthermore, the expression profiles were quantified across organs under different nutrient stresses. Comprehensive characterization of *BnaAMT* genes will provide a foundation for building programs to improve N management and maintain productivity in N-deficient soil for this critical oil crop species.

The 20 rapeseed *BnaAMT* genes distributed over twelve chromosomes and five random chromosomes are at least twice as many as the six in *Arabidopsis* [4,12,13], eight in maize [37], or twelve in rice [32]. Phylogenetic analysis clustered all of the AMT proteins from multiple species into four distinct subfamilies: AMT1, AMT2, AMT3, and AMT4. However, the 20 BnaAMTs in rapeseed only fell into the AMT1 (14 AMT proteins) and AMT2 (6 AMT proteins) subfamily clusters, which is similar to *Arabidopsis* AMT family proteins (Figure 2). Interestingly, each *Arabidopsis AMT* gene exists as a single copy, whereas each *BnaAMT* gene fell into homologous clusters of 2–3 copies (Figure 2). These homologous genes further clustered into single phylogenetic branches without exception (Figure 2). The results herein indicate that duplications of rapeseed *AMT* gene family members resulted primarily from a whole-genome duplication. Thus, polyploidization is likely the main force driving the expansion of the *AMT* gene family in *B. napus*, an allotetraploid plant species [38].

Structural analysis of *BnaAMT* genes revealed that the two subfamilies exhibit divergent exon–intron patterns (Figure 1C). In general, *AMT2* family genes contain more introns than *AMT1* family genes. The *AMT* genes with less intronic sequences, except *BnaAMT1;3a*, were the most highly expressed genes in each of the tested tissues under the applied nutrition treatments. Motif analysis in the MEME application revealed conserved AMT structures across the rapeseed genome (Figure 1C). Specifically, rapeseed *AMT1* family genes have 10–11 nearly uniform motifs. The lone exception is *BnaAMT1;3c*, which lacks Motif2, Motif4, Motif7, and Motif11. In contrast, all the *AMT2* family members in rapeseed lacked Motif 1, Motif 8, and Motif 9, and also contained the *AMT2*-specific Motif 11.

Gene expression profiles may provide essential clues for predicting gene functions. To this end, RNA-seq data were used to investigate *BnaAMT* gene expression levels in diverse tissues of *B. napus* [24,25]. In these observations, 4 of the 20 identified *BnaAMT* genes were minimally expressed across the 12 tested tissues (Figure 3), indicating that these genes fill few functional roles in the tested tissues. On the other hand, *BnaAMT1.1*, *BnaAMT2.1*, and *BnaAMT2;2* family members were expressed across tissues, including in leaves, pericarps, and roots (Figure 3). This did not coincide with previous studies in *Arabidopsis*, which have demonstrated that the members of the *AMT1* clade might preferentially express in roots [11]. In contrast, few rapeseed *AMT* genes exhibited tissue or organ specificity. It is worth mentioning that *BnaAMT1;4b* and *BnaAMT1;4c* were most highly expressed in the young tissues of rapeseed, such as buds and new pistils (Figure 3), indicating that these genes might play roles in rapeseed flower and bud development.

It is generally considered that AMT products mediate ammonium uptake, which has been verified for *AtAMT1;1* by expression under low-NH_4_^+^ conditions in the yeast mutant 31019b, which lacks three ammonium transporter homologous genes known as Meps [29]. Under low-ammonium supply conditions, 31019b mutants harboring *AtAMT1;1* can sufficiently restore ammonium uptake for cellular proliferation. Beyond *AtAMT1;1*, the other *Arabidopsis* AMTs have also restored 31019b growth under low-ammonium conditions [4]. In recent years, the 31019b yeast mutant has been widely applied for functional complementation studies of homologous *AMT* genes in several plant species [39,40,41,42]. For example, in the present study, four of the five rapeseed AMT proteins selected for testing proved capable of restoring 31019b growth under low-ammonium conditions, with *BnaAMT2;2a* being the lone exception (Figure 8). Therefore, it is reasonable to conclude that rapeseed *AMT* genes also function primarily in ammonium uptake.

In plants, *AMT* expression levels are often regulated by the status of multiple nitrogen compounds [4,34,43,44,45]. In the present study, relative to transcription under N deficiency conditions, all rapeseed *AMT2* family members (except *BnaAMT2;2a*) were repressed in young or old leaves subjected to NH_4_^+^ or NO_3_^−^ addition, while expression in roots was generally induced by either NH_4_^+^ and NO_3_^−^ application (Figure 5). Meanwhile, expression of *AMT1* family genes was also enhanced to varying degrees by resupply of either NH_4_^+^ or NO_3_^−^, which is consistent with a previous report that *AMT* transcript levels are subjected to control by NO_3_^−^ availability [34]. Several *AMT* genes, such as *BnaAMT1;1a* and *BnaAMT1;2a*, responded more to resupply with NH_4_^+^ than with NO_3_^−^, especially in old leaves (Figure 5). This result is inconsistent with reports that transcription of *AMT1.1* in *Arabidopsis* is downregulated when NH_4_^+^ is resupplied to N-deficient plants [12]. However, the transient responses observed herein for rapeseed genes such as *BnaAMT1;1a*, *BnaAMT1;2a*, *BnaAMT1;3a*, and *BnaAMT2;2a* indicate that NH_4_^+^ signaling might spike during early phases of N resupply.

With NH_4_^+^ being the preferred N source for plants suffering from N starvation, N deficiency has been noted to strongly induce *AMT1.1* and *AMT1.3* expression in *Arabidopsis*. In comparison, the transcription level of *AMT1.2* has largely been unaffected by N deficiency [12]. In our study, the expression of 11 rapeseed *AMT* genes was significantly enhanced by low-N conditions in leaves or roots, with *BnaAMT1;1b* and *BnaAMT1;1c* being most notable (Figure 5). In realistic conditions, rapeseed planted in fields may suffer from various nutrient deficiencies. In the present study, a portion of the identified *BnaAMTs* exhibited transcriptional responses to several nutrient deficiencies. Particularly noteworthy was the observation that the expression of *BnaAMT1;3c* in the roots increased across all nutrient-deficient conditions, except for N deficiency (Figure 5). This is consistent with previous reports concerning *Pht* family genes in rapeseed [46], which also demonstrated that the expression of rapeseed *AMTs* appears to be involved in mineral nutrient homeostasis and crosstalk among ion signals in response to multiple nutrient stresses.

Both waterlogging and drought stress are also common limiting factors for rapeseed production. Previous studies have found that interactions between waterlogging and fertilizer applications affect rapeseed productivity. In detail, applying N fertilizer has been noted to alleviate the effects of waterlogging stress on rapeseed growth and development, while waterlogging stress can also influence N metabolism [47]. Among our studies, several plant *AMT* genes are affected by drought or waterlogging stress. For example, *AMT2* family members have been significantly enhanced by rehydration after 14 days of drought stress (Figure 6C). In addition, several rapeseed *AMT* genes have also been reported as being influenced by waterlogging stress in both young and old leaves (Figure 7). Previous studies have found that plant *AMTs* could be involved in responses to stress. For example, overexpression of the *Puccinellia tenuiflora* gene *PutAMT1;1* in *Arabidopsis* significantly improves salt tolerance during the early root growth stage after seed germination. This suggests that ammonium transport might alleviate ammonia toxicity caused by salt stress [19]. Overall, upregulation of *AMTs* in response to waterlogging or drought stress suggests that these genes might alleviate stress by improving N uptake and metabolism under stress conditions or by avoiding ammonia toxicity possibly caused by stress through transport across plant tissues.

## 5. Conclusions

In summary, this study includes a comprehensive identification and analysis of *AMT* gene family members in rapeseed. A total of 20 *BnaAMT* genes were identified in the rapeseed genome, each of which was then subjected to bioinformatic and expression profile analyses to reveal their potential functions. The results indicate that *BnaAMT* genes are actively involved in regulating rapeseed plant growth, development, and responses to nutrient deficiency and stresses brought on by drought or waterlogging stress. These results provide a solid foundation for additional functional studies of *BnaAMT* genes and their contributions to stress tolerance in rapeseed, which can be applied to improving crop performance under diverse conditions.

## Figures and Tables

**Table 1:** Information about *AMT* family genes in rapeseed.

Gene Name	Gene Locus	Length of CDS (bp)	No. of Amino Acids (a.a.)
*BnaAMT1;1a*	BnaC08g08610D	1512	503
*BnaAMT1;1b*	BnaC06g11810D	1512	503
*BnaAMT1;1c*	BnaA05g35560D	1512	503
*BnaAMT1;2a*	BnaCnng01740D	1536	511
*BnaAMT1;2b*	BnaA09g00320D	1539	512
*BnaAMT1;3a*	BnaA07g05760D	1515	504
*BnaAMT1;3b*	BnaUnng02430D	1482	493
*BnaAMT1;3c*	BnaA01g23190D	999	332
*BnaAMT1;4a*	BnaC07g41470D	1530	509
*BnaAMT1;4b*	BnaC01g09770D	1506	501
*BnaAMT1;4c*	BnaA01g08220D	1509	502
*BnaAMT1;5a*	BnaA03g37270D	1428	475
*BnaAMT1;5b*	BnaC03g42390D	1428	475
*BnaAMT1;5c*	BnaC03g74280D	1428	475
*BnaAMT2;1a*	BnaA05g06450D	1470	489
*BnaAMT2;1b*	BnaC04g07100D	993	330
*BnaAMT2;1c*	BnaC04g07090D	1344	447
*BnaAMT2;2a*	BnaCnng62050D	1107	368
*BnaAMT2;2b*	BnaC04g56650D	1497	498
*BnaAMT2;2c*	BnaA04g21900D	1467	488

## Data Availability

The AMT protein sequences of *B. rape*, *B. oleracea*, and *B. juncea* were collected from BRAD (http://brassicadb.org/brad/ (accessed on 13 February 2023)) and the genome and protein sequences of *B. napus* were downloaded from Genoscope (http://www.genoscope.cns.fr/brassicanapus/ (accessed on 13 February 2023)). All data generated or analyzed in this study were included in this published article and its additional files. The materials are available upon request by contacting the corresponding author.

## Supplementary Materials

The following supporting information can be downloaded at: https://www.mdpi.com/article/10.3390/genes14030658/s1, Table S1: Specific primers of the rapeseed AMT genes used in qRT-PCR assays; Table S2: Primers used in the yeast complementation experiments; Table S3: Primers used in the subcellular localization experiments.
