# Supplementary material for: Genome-Wide Identification and Characterization of Ammonium Transporter (AMT) Genes in Rapeseed (Brassica napus L.)
Source: Genes (Basel). 2023 Mar 6;14(3):658. doi: 10.3390/genes14030658 (PMC10048622; doi:10.3390/genes14030658)
Supplement: Supplementary file 1 [file genes-14-00658-s001.zip › Supplementary Table S1.pdf]

Supplementary Table S1 Specific primers of rapeseed *AMT* genes used in qRT-PCR assays.

Gene name	Gene ID	Forward	Reverse
BnaAMT1;1a	BnaC08g08610D	CGTTCCGTACCGAAGGTGAT	CGATTAACGCACCCCAGAGT
BnaAMT1;1b	BnaC06g11810D	CTATAACCGGTGGCTGCTCG	TTGAGTTTCTCCGCGAGCTT
BnaAMT1;1c	BnaA05g35560D	TATAACCGGTGGCTGTTCGG	TTGAGTTTCTCCGCGAGCTT
BnaAMT1;2a	BnaCnng01740D	GCCTACGCGTACAACGATGA	GCAGTCAAGGTCGGTGTAGG
BnaAMT1;2b	BnaA09g00320D	GATTGCTAGGTGGCTTTGCG	GGCGAGGAAGTTAAACCCGA
BnaAMT1;3a	BnaA07g05760D	TCACGGTGGTTTTGCTTATATGT	TAAACGCGAGGAGGAGTAAC
BnaAMT1;3b	BnaUnng02430D	TGGCCGGTTCGAGAAAAGTG	CCAGGGTTGAAACTATTGGCA
BnaAMT1;3c	BnaA01g23190D	AGCTCTAACTTGCTCTGCCG	TAACAGTGCCTAGTTGGCCG
BnaAMT1;4a	BnaC07g41470D	GGGGGATCATTTTCACTGGTT	TCCTCCCATAAACAGCCCAA
BnaAMT1;4b	BnaC01g09770D	GTTGACGACTCTGTTCGGGA	GGTTCAACAACCGAGCAACC
BnaAMT1;4c	BnaA01g08220D	GACGTTGGTTGTACTTGGTACG	TCCGTAGAACGAGCTTCCC
BnaAMT1;5a	BnaA03g37270D	TACATGGAGGCCCAGGAAGG	GGCACTAACCCATCCCACAA
BnaAMT1;5b	BnaC03g42390D	CAGCTACACGGAGGCCC	TCCCATTGTGGCACTAACCC
BnaAMT1;5c	BnaC03g74280D	CAGCTACACGGAGGCCC	TCCCATTGTGGCACTAACCC
BnaAMT2;1a	BnaA05g06450D	GCCGGAGCTTACGATGCTAAT	GCATGCTTTGTAGACCGACC
BnaAMT2;1b	BnaC04g07100D	ATGATCATTCACAAGAAATCCACTC	AACAAGCCTGTCAGTAATCCAC
BnaAMT2;1c	BnaC04g07090D	CACCACTTGTGTGACTTGTCT	TGACTCTGGTTTCTTGAGAGGT
BnaAMT2;2a	BnaCnng62050D	TGATCCAAACATGGGCAGCTA	AGGGTGGAGATAATTCACCTGTT
BnaAMT2;2b	BnaC04g56650D	GATCATCTTGCCGAGTGTTCC	CTGGCATGCTTTGTAGACCG
BnaAMT2;2c	BnaA04g21900D	AGAAATGGGCCGTGAACTCC	CCCAGAAAGGCAAAAGCTCG
Actin7	BnaA03g55890D	CTGGAATTGCTGACCGTATGAG	ATCTGTTGGAAAGTGCTGAGGG
