# Supplementary material for: Genome-Wide Identification and Characterization of Ammonium Transporter (AMT) Genes in Rapeseed (Brassica napus L.)
Source: Genes (Basel). 2023 Mar 6;14(3):658. doi: 10.3390/genes14030658 (PMC10048622; doi:10.3390/genes14030658)
Supplement: Supplementary file 1 [file genes-14-00658-s001.zip › Supplementary Table S2.pdf]

Supplementary Table S2 Primers used in the yeast complementation experiments

Gene name	Gene ID	Forward
BnaAMT1;1b-HindIII-F		CCCaagcttCCTAGGATGTCGGGATCTTTATCTTG
BnaAMT1;1b-XbaI-R		GGCGCGCCtctagaAACAGAAGTGGTAGTAACAC
BnaAMT1;1c-HindIII-F		CCCaagcttCCTAGGATGTCGGGATCTTTATCTTG
BnaAMT1;1c-XbaI-R		GGCGCGCCtctagaAACAGAAGTGGTAGTAACAC
BnaAMT1;4a-KpnI-F		GGggtaccCCTAGGATGGCGTCGTCGACAATCTC
BnaAMT1;4a-XbaI-R		GGCGCGCCtctagaAAGAGCTGGAGGATCAGAAC
BnaAMT1;5a-HindIII-F		CCCaagcttCCTAGGATGTCTGGAGCTATAACATG
BnaAMT1;5a-XbaI-R		GGCGCGCCtctagaAAGAGCTGGAGGATCAGAAC
BnaAMT2;2a-HindIII-F		CCCaagcttATGGCCGGAGCTTACGGTTC
BnaAMT2;2a-XbaI-F		GCtctagaATAATGATATAAGAAATACA
