# Supplementary material for: Genome-Wide Identification and Characterization of Ammonium Transporter (AMT) Genes in Rapeseed (Brassica napus L.)
Source: Genes (Basel). 2023 Mar 6;14(3):658. doi: 10.3390/genes14030658 (PMC10048622; doi:10.3390/genes14030658)
Supplement: Supplementary file 1 [file genes-14-00658-s001.zip › Supplementary Table S3.pdf]

**Supplementary Table S3 Primers used in the subcellular localization experiments**

| Gene name | Gene ID | Forward |
|---|---|---|
| *BnaAMT1;1b-HindIII-F* | | cagtCGTCTCacaacatgtcgggatctttatcttg |
| *BnaAMT1;1b-XbaI-R* | | cagtCGTCTCatacaaacagaagtggtagtaacac |
| *BnaAMT1;1c-HindIII-F* | | CCCaagcttCCTAGGATGTCGGGATCTTTATCTTG |
| *BnaAMT1;1c-XbaI-R* | | GGCGCGCCtctagaAACAGAAGTGGTAGTAACAC |
| *BnaAMT1;4a-KpnI-F* | | GGggtaccCCTAGGATGGCGTCGTCGACAATCTC |
| *BnaAMT1;4a-XbaI-R* | | GGCGCGCCtctagaAAGAGCTGGAGGATCAGAAC |
| *BnaAMT1;5a-HindIII-F* | | CCCaagcttCCTAGGATGTCTGGAGCTATAACATG |
| *BnaAMT1;5a-XbaI-R* | | GGCGCGCCtctagaAAGAGCTGGAGGATCAGAAC |
| *BnaAMT2;2a-HindIII-F* | | CCCaagcttATGGCCGGAGCTTACGGTTC |
| *BnaAMT2;2a-XbaI-F* | | GCtctagaATAATGATATAAGAAATACA |
